# Dendritic cells respond to nasopharygeal carcinoma cells through annexin A2-recognizing DC-SIGN

**DOI:** 10.18632/oncotarget.2700

**Published:** 2014-11-06

**Authors:** Pin-Zhir Chao, Ming-Shium Hsieh, Chao-Wen Cheng, Tin-Jui Hsu, Yun-Tien Lin, Chang-Hao Lai, Chen-Chung Liao, Wei-Yu Chen, Ting-Kai Leung, Fei-Peng Lee, Yung-Feng Lin, Chien-Ho Chen

**Affiliations:** ^1^ Graduate Institute of Clinical Medicine, College of Medicine, Taipei Medical University, Taipei, Taiwan; ^2^ Department of Otolaryngology, Shuang-Ho Hospital, New Taipei, Taiwan; ^3^ Department of Orthopedics, En Chu Kong Hospital, New Taipei, Taiwan; ^4^ School of Medical Laboratory Science and Biotechnology, College of Medical Science and Technology, Taipei Medical University, Taipei, Taiwan; ^5^ Proteomics Research Center, National Yang-Ming University, Taipei, Taiwan; ^6^ Department of Pathology, Wan Fang Hospital, Taipei, Taiwan; ^7^ Department of Radiology, School of Medicine, College of Medicine, Taipei Medical University, Taipei, Taiwan; ^8^ Department of Otolaryngology, Head and Neck Surgery, Wan-Fang Medical Center, Taipei, Taiwan

**Keywords:** dendritic cell, DC-SIGN, NPC, annexin A2, IL-10, immunosuppression

## Abstract

Dendritic cells (DCs) play an essential role in immunity and are used in cancer immunotherapy. However, these cells can be tuned by tumors with immunosuppressive responses. DC-specific intercellular adhesion molecule 3-Grabbing Nonintegrin (DC-SIGN), a C-type lectin expressed on DCs, recognizes certain carbohydrate structures which can be found on cancer cells. Nasopharyngeal carcinoma (NPC) is an epithelial cell-derived malignant tumor, in which immune response remains unclear. This research is to reveal the molecular link on NPC cells that induces the immunosuppressive responses in DCs. In this article, we report identification of annexin A2 (ANXA2) on NPC cells as a ligand for DC-SIGN on DCs. N-linked mannose-rich glycan on ANXA2 may mediate the interaction. ANXA2 was abundantly expressed in NPC, and knockdown of ANXA2 suppressed NPC xenograft in mice, suggesting a crucial role of ANXA2 in NPC growth. Interaction with NPC cells caused DC-SIGN activation in DCs. Consequently DC maturation and the proinflammatory interleukin (IL)-12 production were inhibited, and the immunosuppressive IL-10 production was promoted. Blockage of either DC-SIGN or ANXA2 eliminated the production of IL-10 from DCs. This report suggests that suppression of ANXA2 at its expression or glycosylation on NPC may improve DC-mediated immunotherapy for the tumor.

## INTRODUCTION

Dendritic cells (DCs) are essential antigen-presenting cells (APCs) recognizing pathogens and tumors. An effective response to tumors requires innate and adaptive immunity coordinated by DCs, key regulators of T cell-mediated immune responses. Recently the use of DCs becomes a major focus in cancer immunotherapy; however, many DC therapies resulted in limited clinical benefits [1]. DCs can polarize naïve T cells toward Th1 or Th2 pathway, which plays an important role on the way to specify immune responses [2]. Two receptor families on DCs are involved in this process. Toll-like receptors recognize common pathogen-associated molecules, while C-type lectins are receptors recognizing glycosylated antigens [3]. Toll-like receptors signal to promote DC maturation and induce the production of Th1-polarizing cytokines such as IL-12. In contrast, immature DCs strongly express DC-specific intercellular adhesion molecule-3-grabbing nonintegrin (DC-SIGN; CD209; UniProtKB: Q9NNX6), a kind of C-type lectins [4]. DC-SIGN is expressed on myeloid, dermal and monocyte-derived DCs and functions through calcium-dependent carbohydrate binding [5]. The engagement of DC-SIGN by mannose- or fucose-containing oligosaccharides can lead to an altered Toll-like receptor signaling, resulting in a Th2 response [5, 6]. DC-SIGN signaling increases IL-10 production which is critical for proper immunosuppression [7, 8].

Tumors can escape the immune system by interfering with DC activities including DC maturation and certain cytokine productions [9]. Glycosylation changes associated with tumors play an important role on inactivation of DCs [10]. Recent studies demonstrated that mannose- and fucose-expressing ligands on microbes induced different effects on the function of DCs [11]. Mannose-expressing ligands triggers an inflammatory response mediated by DCs, whereas fucose expressing ligands suppress the ability of DCs to produce the inflammatory cytokine such as IL-12, enhancing the production of IL-10. Other findings indicated that tumor-related glycol-forms of certain proteins such as carcinoembryonic antigen (CEA), Mac-2–binding protein (Mac-2BP) and mucin (MUC) 1 are specific ligands for DC-SIGN [12-14]. They instruct DCs to drive Th2-mediated responses that, unlike Th1 responses, do not contribute to tumor eradication [15].

Nasopharyngeal carcinoma (NPC) is the most common tumor originating in the nasopharynx and differs from other head and neck cancers. It is most commonly found in East Asia and Africa with viral, dietary and genetic factors related in its causation. Recurrent NPC represents a small proportion of head-and-neck cancers with a unique set of patho-clinical characteristics [16]. Although the outcomes in patients with primary NPC have improved due to advances in radiotherapy and chemotherapy, the management of recurrent NPC remains a clinical dilemma because of an incomplete understanding of its pathologic mechanism. Alternatively, the patients may be treated with immunotherapy which is being studied intensively. Recent studies reported the presence of DCs in NPC biopsies, and DCs are often found inside the malignant cell nests, suggesting a potential DC-based immunotherapy for NPC [17-19]. In addition, a couple of DC-SIGN genotypes were found to be associated with NPC risk [20]; however, the role of DCs in NPC remains unclear.

In the present study, we identified annexin A2 (annexin II; ANXA2; UniProtKB: P07355) on NPC cells as a DC-SIGN ligand. ANXA2 can form a heterotetramer with p11 (S100A10) and associate with plasma membrane [21, 22]. Its deregulation was found in a variety of tumors, including NPC, associated with the occurrence, invasion and metastasis of those tumors [23]. ANXA2 is a glycoprotein with at least one site of asparagine (N)-linked biantennary mannosyl residues [24].

We hypothesize that NPC cells escape from the surveillance of immune system by modifying DC-SIGN ligand ANXA2, resulting in immunosuppressive cytokine production in DCs through DC-SIGN signaling. Indeed, we identified ANXA2 as a target for DC-SIGN. The glycan modification of ANXA2 on NPC cells was involved in their interaction, following which can alter DC activities toward a Th2 response.

## RESULTS

### Maturation and cytokine production of MDDCs were altered by NPC cells

**Figure 1:**
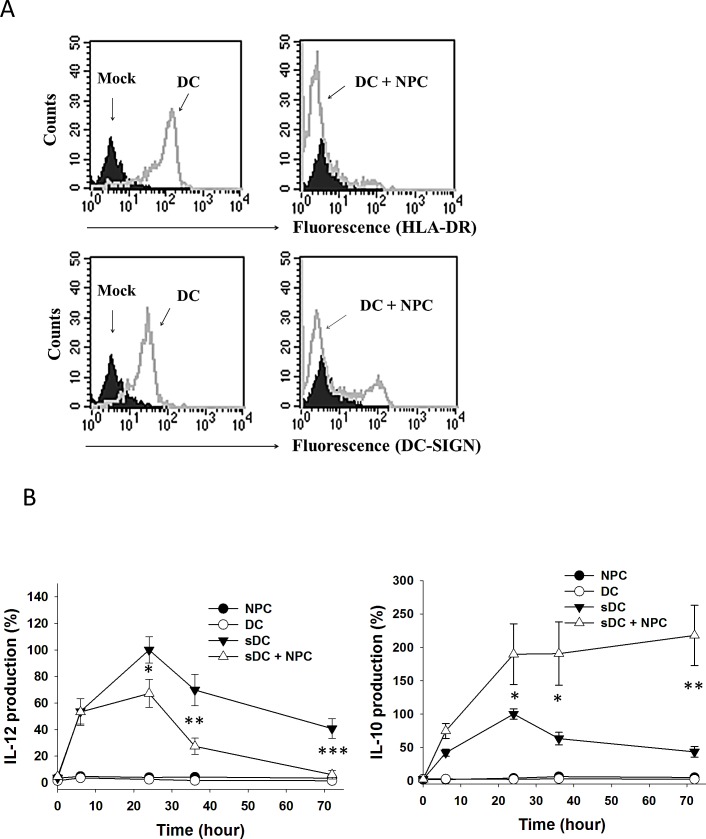
Maturation and cytokine production of DC co-cultured with NPC cells A. Flow cytometry on DC showing HLA-DR and DC-SIGN expression. DCs without labelling (mock) were shown in black. Cells labelled with HLA-DR-FITC or DC-SIGN-FITC were shown in gray curves. Left panels, LPS-stimulated DC; right panels, LPS-stimulated DC co-cultured with NPC cells. B. IL-12 (left panel) and IL-10 (right panel) production of DC co-cultured with NPC cells in various conditions and time periods. The cytokine production levels from LPS-stimulated DC at 24h were set as control (100%). At least three sets of independent experiments were performed. * p<0.05, ** p<0.01, *** p<0.001.

To test the effect of NPC cells on DCs, we cultured MDDCs with TW01 NPC cells. With LPS stimulation, the MDDCs expressed a high level of the maturation marker HLA-DR, a subunit of antigen presenting complex, and DC-SIGN (Fig. 1A, left panel). Upon co-cultured with NPC cells, only a small portion of MDDCs expressed HLA-DR and DC-SIGN, suggesting an inhibitory effect of NPC cells on DC maturation (Fig. 1A, right panel). We then collected the culture media and determined the concentration of proinflammatory cytokine IL-12 and immunosuppressive cytokine IL-10 by ELISA. Within 72 h, LPS-stimulated MDDCs produced IL-12 and IL-10 consistently with highest levels around 24^th^ h (Fig. 1B). Under NPC-co-cultured condition, IL-12 production in the MDDCs was gradually decreased after 24 h (Fig. 1B, left panel), while IL-10 kept increasing afterward (Fig. 1B, right panel). These data indicated that NPC cells interfered with DC maturation and directed DC cytokine production toward immunosuppressive responses which could be mediated through DC-SIGN.

### NPC cells expressed DC-SIGN ligand(s)

**Figure 2:**
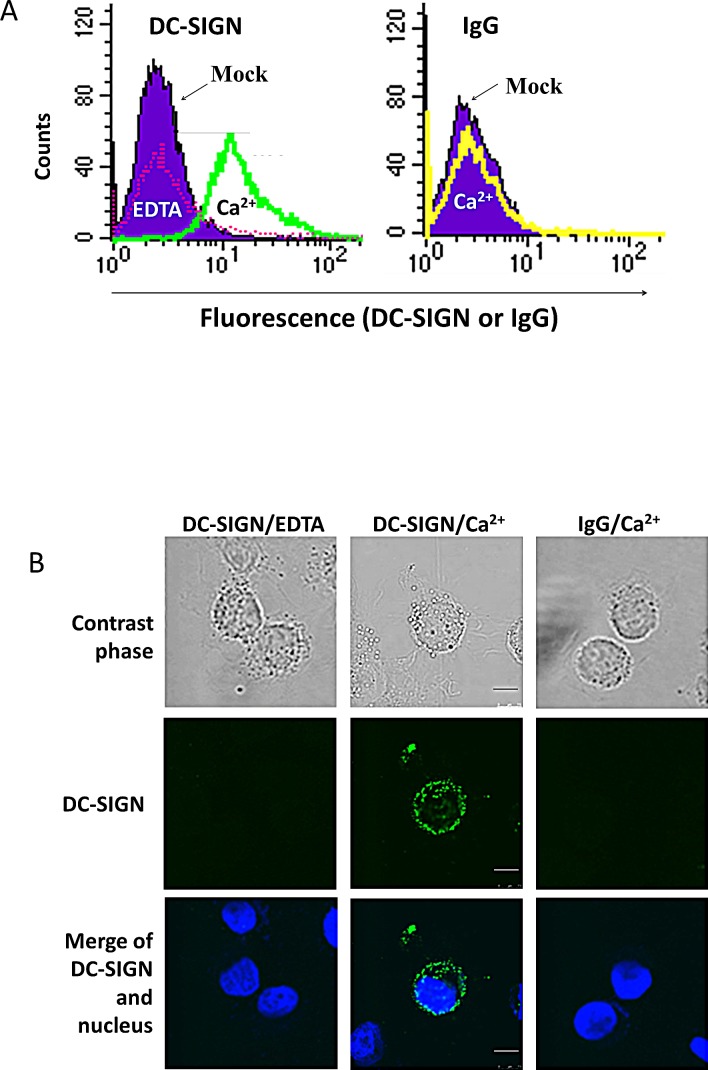
Expression of DC-SIGN ligand(s) on NPC cell surface A. Flow cytometry on NPC cells treated with DC-SIGN recombinant protein and labelled with DC-SIGN antibody and FITC. NPC cells without treatment were shown in purple. The DC-SIGN-treated and -labelled NPC cells in the presence of EDTA and Ca2+ were shown in pink and green curves, respectively (left panel). The DC-SIGN-treated but IgG-labelled NPC cells were shown in a yellow curve (right panel). B. Confocal fluorescent images showing DC-SIGN binding on NPC cell surface. The nuclei were stained with DAPI. Three independent experiments were performed. Scale bar, 10 μm.

When NPC cells were treated with DC-SIGN recombinant protein and labelled with DC-SIGN antibody and FITC in the presence of Ca^2+^, the cells became fluorescent in flow cytometry (Fig. 2A). This result was not seen in the presence of EDTA, a Ca^2+^ chelator, or replacing DC-SIGN with IgG, indicating a specific binding of DC-SIGN on NPC cells. Consistent results were seen in the immunofluorescence staining (Fig. 2B). These data suggest that NPC cells express DC-SIGN ligand(s) on their cell membrane.

**Figure 3:**
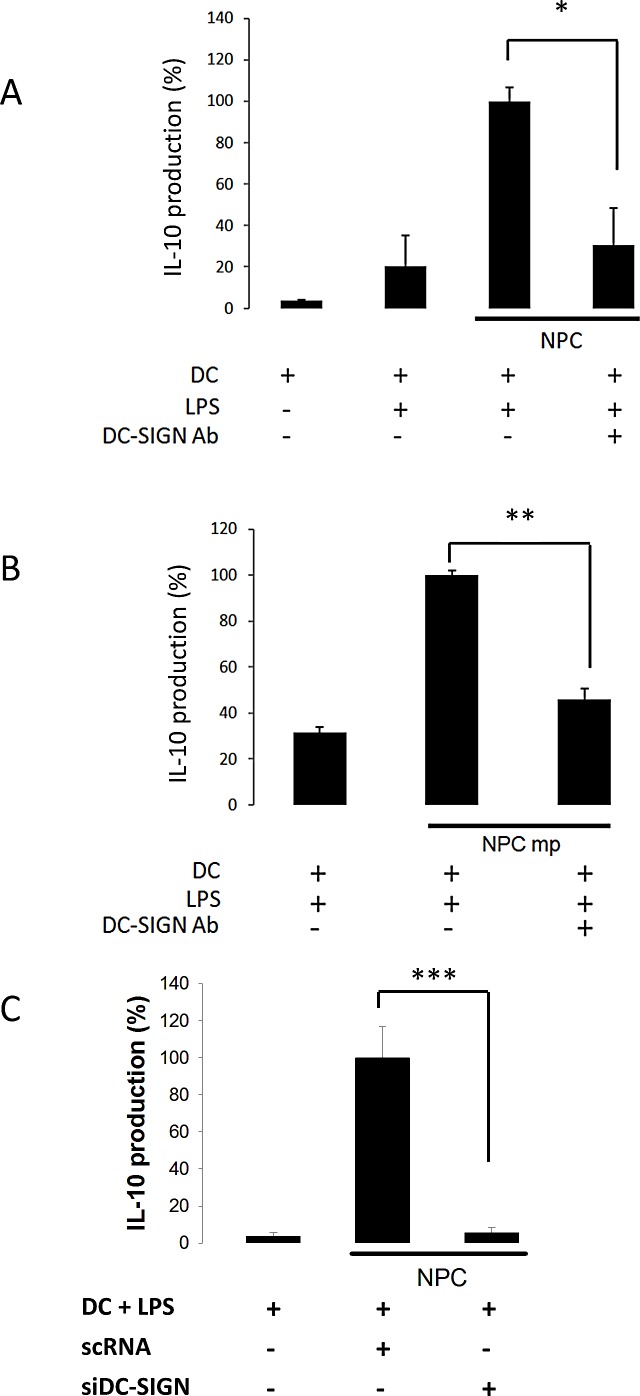
NPC-regulated IL-10 production of DC through DC-SIGN A. Reduced IL-10 production from anti-DC-SIGN antibody-treated DC co-cultured with NPC cells. B. Reduced IL-10 production from anti-DC-SIGN antibody-treated DC incubated with NPC membrane proteins (mp). C. Reduced IL-10 production from DC-SIGN-silenced DC co-cultured with NPC cells. The IL-10 level from LPS- and NPC-stimulated DC was set as 100% control. Three sets of each experiment were performed. * p<0.05, ** p<0.01, *** p<0.001.

DC-SIGN antibody and siRNA were applied to further determine the dependence of DC-SIGN for MDDC and NPC cell interactions. As shown on Fig. 3, DC-SIGN antibody was dramatically inhibited both the IL-10 productions promoted by NPC whole cells (Fig. 3A) and their membrane proteins (Fig. 3B) after 24 h of culture. Likewise, the siRNA completely inhibited NPC-promoted IL-10 production of MDDCs (Fig. 3C). These results demonstrate a specific interaction of NPC cells with DCs through DC-SIGN.

### DC-SIGN bound ANXA2 which was highly expressed in NPC

**Figure 4:**
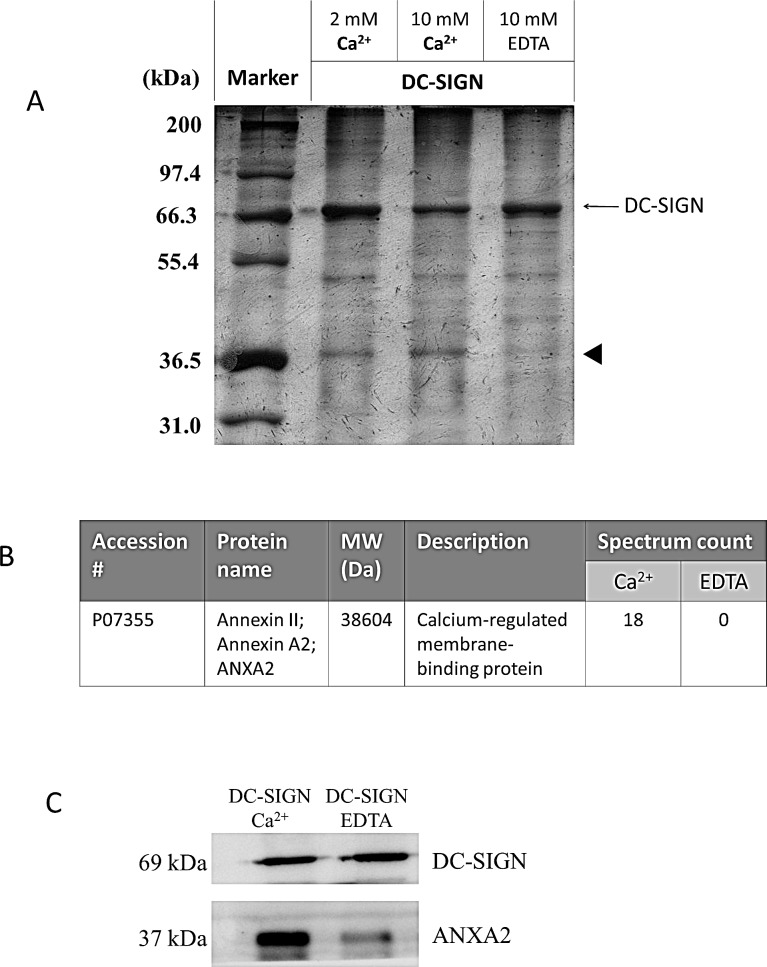
Immunoprecipitation of DC-SIGN-binding proteins on NPC cell membranes NPC cell membrane proteins were incubated with human IgG-Fc-fused DC-SIGN recombinant protein at various conditions and precipitated using protein A beads. A. SDS-PAGE of immunoprecipitates. Proteins were analyzed in a 12% SDS-PAGE with silver staining. Indicated proteins (arrow head) were extracted and identified by LC MS/MS. B. Identification of ANXA2 as a DC-SIGN-binding protein. C. Immunoblotting of DC-SIGN precipitates. Proteins were analyzed by immunoblotting with antibodies against DC-SIGN and ANXA2. Three independent experiments were performed.

DC-SIGN-Fc recombinant protein was used to precipitate NPC membrane proteins in the presence of Ca^2+^ or EDTA. On the silver-stained gel, a protein species with a molecular weight around 37 kDa was found in the presence of Ca^2+^ but not EDTA (Fig. 4A). After LC/MS/MS analyses, ANXA2 was identified as a significant interacting protein of DC-SIGN (Fig. 4B). Western blotting with DC-SIGN and ANXA2 antibodies on the same precipitates confirmed that ANXA2 from NPC cells bound DC-SIGN (Fig. 4C).

**Figure 5:**
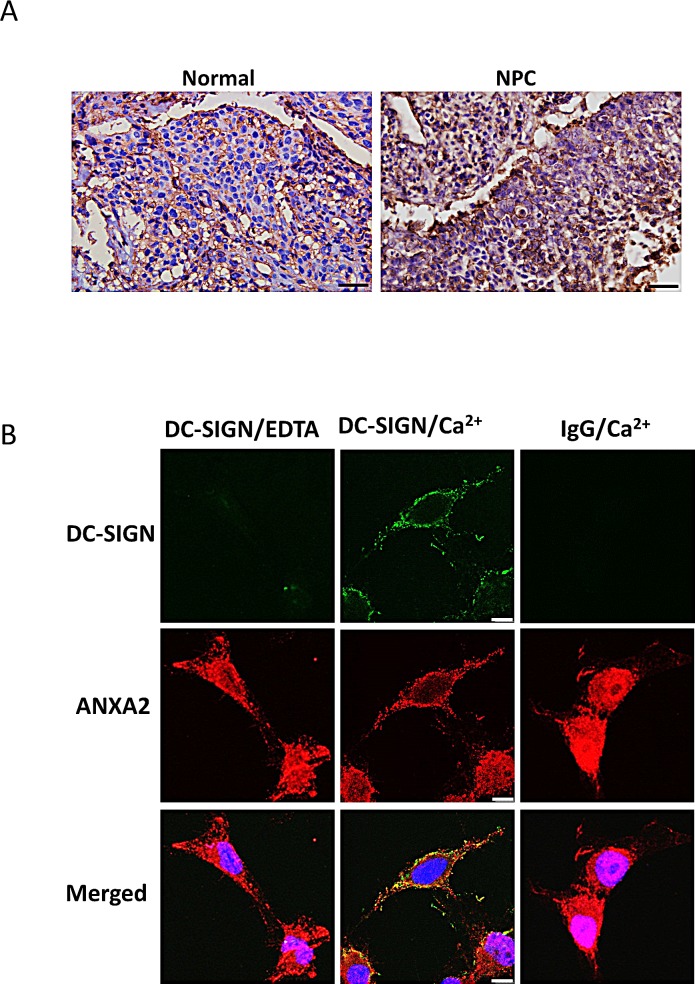
Expression and localization of ANXA2 in NPC A. Immunohistochemistry of human normal and NPC tissues showing the expression of ANXA2. DAB and hematoxylin were used for the staining. Scale bar, 50 μm. B. Confocal fluorescent images showing the co-localization of Annexin A2 and DC-SIGN on NPC cells. Anti-Human IgG-Fc-FITC or anti-rabbit IgG-DyLight 594 antibody was used. Three sets of independent experiments were performed. Scale bar, 10 μm.

We then examined the expression pattern of ANXA2 in NPC. Immunohistochemistry data showed a higher ANXA2 level in patient's NPC than in normal nasopharyngeal tissue (Fig. 5A). High expression level of ANXA2 was also found in TW01 NPC cells (Fig. 5B). Fluorescent images of the cells showed colocalization of ANXA2 and the exogenous DC-SIGN-Fc on NPC cell surface in the presence of Ca^2+^. Taken together, ANXA2 interacts with DC-SIGN and may play an important role in the DC-mediated immunosuppression.

### NPC cell activities were reduced when ANXA2 was knocked down

**Figure 6:**
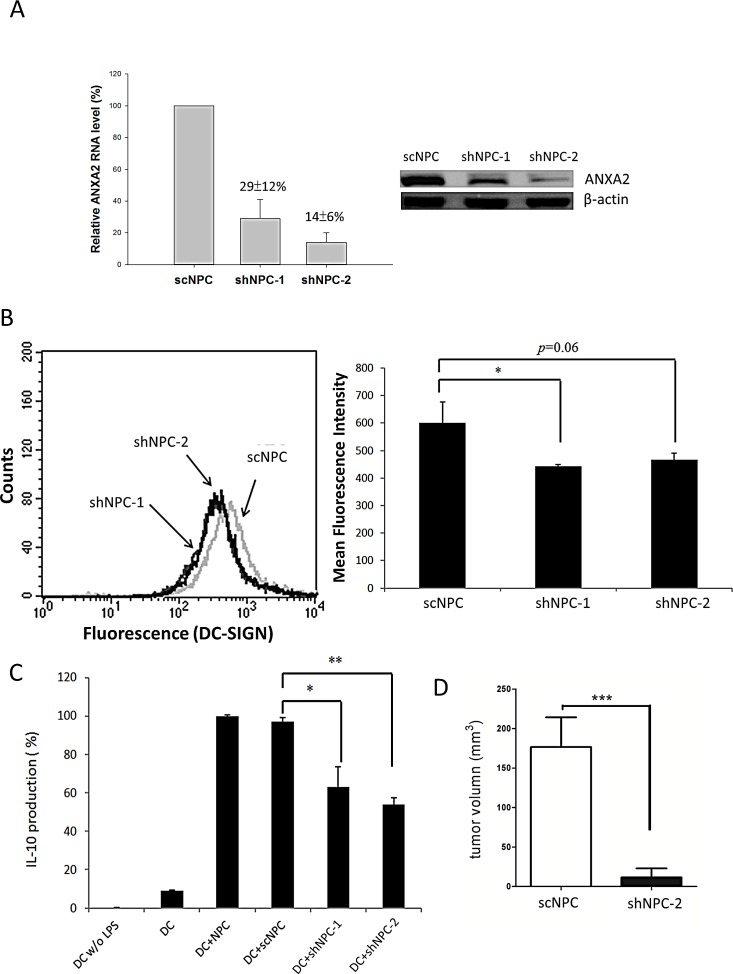
Reduction of NPC activities by ANXA2 knockdown A. Knockdown of ANXA2 in NPC cells by shRNAs. The mRNA levels of NPC cells harboring a scramble shRNA (scNPC) or ANXA2 shRNA (shNPC-1 or shNPC-2) were determined by real-time PCR (left panel). The protein levels of the shRNA-harboring NPC cell lines were assayed by western blotting (right panel). B. Flow cytometry showing the reduction of DC-SIGN binding capacity of NPC cells with ANXA2 knockdown. The shRNA-harboring NPC cells were incubated with DC-SIGN-Fc recombinant protein and labelled with anti-DC-SIGN antibody for flow cytometric analysis (left panel). The statistical results were shown in the right panel. C. ELISA showing reduced IL-10 production from DC co-cultured with ANXA2-knocked down NPC cells. D. Human NPC xenografts in mice. The tumor volume on immunodeficient NSG mice hosting shNPC-2 cells was reduced comparing to those hosting scNPC. Three sets of each experiment were performed. * p<0.05, ** p<0.01, *** p<0.001.

ANXA2 was knocked down by RNA interference to characterize the role of ANXA2 in NPC cells. Both ANXA2 shRNAs knocked down the expression at both mRNA and protein levels in NPC cells (shNPC-1 and shNPC-2) efficiently (Fig. 6A). After treated with DC-SIGN-Fc recombinant protein and anti-human IgG (Fc fragment)-FITC antibody, fluorescent NPC cells were counted in flow cytometry. The mean fluorescent intensity was reduced in both ANXA2-knocked down cell lines (Fig. 6B). The results indicated a specific interaction of ANXA2 and DC-SIGN although the reduction in shNPC-2 cells seemed not significant, which could be due to *in vitro* interferences on the binding capacity of DC-SIGN-Fc. Indeed, both ANXA2-knocked down NPC cell lines were significantly decreased in promoting MDDCs to produce IL-10 (Fig. 6C). When shNPC-2 cells were used as a xenograft in mice, the tumor growth was dramatically inhibited compared to the control mice (Fig. 6D), suggesting a potent antitumor effect of ANXA2 knockdown, which may involve a restoration from DC-mediated immune suppression.

### Certain glycosylation pattern is required for the binding of ANXA2 by DC-SIGN

**Figure 7:**
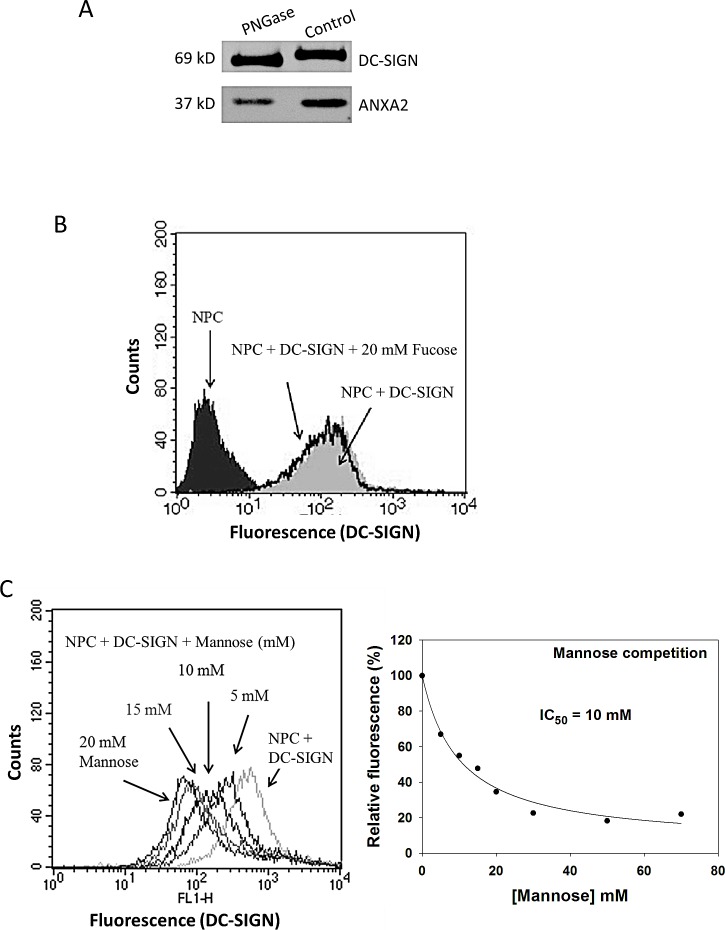
Involvement of glycans on NPC cells in binding DC-SIGN A. Western blotting of DC-SIGN precipitates showing reduced ANXA2 pulldown from NPC cells after glycan digestion by peptide-N-glycosidase (PNGase). B. Flow cytometry on DC-SIGN-bound NPC cells showing no interference of fucose at 20 mM. C. Flow cytometry on DC-SIGN-bound NPC cells showing dose-dependent inhibition by mannose (left panel). Regression plot suggested the 50% inhibition concentration (IC_50_) at 10 mM (right panel). Three independent experiments were performed.

To determine the type of glycan involved in the interaction of ANXA2 and DC-SIGN, NPC cell membrane proteins were treated with PNGase F, an N-glycan-digesting enzyme, and then precipitated with DC-SIGN-Fc. As shown in Fig. 7A, DC-SIGN-Fc bound less ANXA2 with PNGase F treatment than that without treatment, suggesting the involvement of N-linked glycosylation on ANXA2 in NPC. Two monosaccharides, namely fucose and mannose, were then used to compete the binding of DC-SIGN-Fc on NPC cell. Flow cytometry results showed no inhibition of DC-SIGN binding on NPC cells by 20 mM fucose (Fig. 7B). In contrast, mannose inhibited the binding with an IC_50_ of 10 mM (Fig. 7C), suggesting that mannose may constitute an important part in the glycan moiety of ANXA2 on NPC cells.

## DISCUSSION

The use of DCs is a major focus in cancer immunotherapy; however, many attempts resulted in limited clinical outcomes which may be due to DC-SIGN-mediated immunosuppressive responses. In this study, we identified ANXA2 on NPC cells as a ligand for DC-SIGN on DCs. Interaction of ANXA2 and DC-SIGN inhibited DC maturation and promoted immunosuppressive IL-10 production, resulting in NPC outgrowth. We therefore propose that ANXA2 may be used for target therapy on NPC and perhaps other cancers.

ANXA2 is a calcium-dependent, phospholipid-binding protein found on the surface of many cell types [21, 22]. The formation of ANXA2-S100A10 heterotetramer results in the association of the complex with plasma membrane. Recently it was shown that ANXA2 heterotetramer facilitates human papillomavirus-inhibited maturation of Langerhans cell, another type of APCs, inducing immune suppression [25]. Indeed, ANXA2 plays a key role in immune tolerance. In the present study, we further described that DC-SIGN is an interacting partner of ANXA2, and a certain glycosylation pattern on ANXA2 is required for their interactions.

DC-SIGN recognizes certain carbohydrate structures on a variety of proteins. Recent studies of colon cancer revealed interactions of DC-SIGN and a few glycoproteins such as CEA, Mac-2BP and MUC1 on the cancer cell surface [12-14]. Their interactions with DC-SIGN interfere with DC maturation and increase IL-10 production [13, 26], similar to the effects of ANXA2-DC-SIGN interaction in the present report (Fig. 1); however, these proteins vary in the composition of glycans. ANXA2 contains N-linked glycosylation with mannose to interact with DC-SIGN (Fig. 7) that is similar to CEA, while Mac-2BP requires fucose to do so, and MUC1 is far different in O-linked structures.

It seems unexpectedly that ANXA2-knockdown NPC cells still bound DC-SIGN with remaining three quarters of capacity (Fig. 6B). Note that DC-SIGN can bind carbohydrate structures on other proteins. The reduction of 69-86% ANXA2 in the cells (Fig. 6A) removed nearly a quarter of DC-SIGN binding capacity, suggesting that there is about one third of DC-SIGN bound by ANXA2 on NPC cells. In addition, ANXA2 can be distributed in a variety of cellular compartments including cytoplasm, intracellular membranes and external face of the plasma membrane [27]. The reduction of ANXA2 by RNA interference may not decrease its distribution on the cell surface as much as inside the cell. Indeed, another study on hepatoma xenograft in mice demonstrated that the distribution of ANXA2 is mainly localized to the cell membrane in shRNA-bearing group [28]. The binding capacity of DC-SIGN by ANXA2 could be underestimated.

It is striking that the ANXA2-knockdown NPC cells were significantly reduced in promoting IL-10 production in DCs (Fig. 6C) and suppressed in xenograft growth (Fig. 6D). According to other immunohistochemical studies, IL-10 is detected in NPC biopsies [29]. Interestingly, IL-10 is present in the leucocyte infiltrate of the tumor instead of in the NPC cells [30]. Herein our result suggests a substantial involvement of ANXA2 and DC-SIGN interaction in regulating cytokine production of DCs and the progression of NPC. Further studies may be needed to distinguish the effects of exoplasmic from cytoplasmic ANXA2 on tumor growth.

IL-10 is a cytokine associated with immune suppression. It was identified as an inhibitor for other cytokine synthesis and antigen presentation [31]. Inhibitory effect of IL-10 on T cells was mediated mainly through APCs such as DCs [8]. High concentrations of IL-10 promote naïve T cells to differentiate into regulatory T (Treg) cells [32], and Treg cells produce more IL-10 in positive feedback regulation [33]. These mechanisms decrease production of pro-inflammation helper T cells to inhibit immune responses. It seems likely that cancer cells, including NPC cells, commonly regulate immune suppression by stimulating IL-10 production in DCs. Supporting evidence was demonstrated in the present study. We used LPS to activate Toll-like receptor pathway which induced both IL-12 and IL-10 production in MDDCs [7] (Fig. 1B); however, when co-cultured with NPC cells, MDDCs increased IL-10 but decreased IL-12 production, supporting an immunosuppressive response.

Other molecules may be involved in the interactions of ANXA2 and DC-SIGN. An example is C1q, an ANXA2 receptor initiating the classical complement pathway to facilitate phagocytosis [34] and a DC-SIGN partner modulating the differentiation of MDDCs [35]. Future works may be required for us to reveal more details of these interactions and provide practicable advice in cancer immunotherapy.

## MATERIALS AND METHODS

### Animals

To monitor the NPC growth *in vivo*, immunodeficient NOD/SCID/IL2r-γnull (*NSG*) mice were engrafted with TW01 NPC cell lines. A total of 2×10^7^ cells in 100 μl of PBS was transplanted subcutaneously (s.c.) in the mouse abdomen. After 14 days, the tumor size was measured by Short*Short*Long/2 (mm^3^). Totally 7 mice in each group were examined. All the animal procedures were approved by the Laboratory Animal Center in Taipei Medical University.

### Preparation of cells

NPC cell line TW01 is Epstein-Barr virus negative and derived from the NPC of a Taiwanese patient [36]. It is categorized as WHO type I, squamous cell carcinoma. The cells were maintained in DMEM containing 10% FBS, 100 units/ml penicillin, and 100 μg/ml streptomycin at 37°C with 5% CO_2_.

Monocyte-derived DCs (MDDCs) were prepared from primary monocytes (CD14^+^) separated from the peripheral blood mononuclear cells of healthy donors by magnetic beads conjugated with anti-CD14 antibody (MACS, Germany). Totally nine healthy subjects were recruited in the study. Primary cells were maintained in RPMI 1640 medium containing 10% FBS (Biological Industries, Israel), 100 units/ml penicillin, and 100 μg/ml streptomycin at 37°C with 5% CO_2_. Monocytes were cultured in a medium containing IL-4 (25 ng/ml) and granulocyte monocyte colony–stimulating factor (50 ng/ml) for six to seven days to be differentiated into immature MDDCs. The cultured MDDCs were over 90% survival rate and purity to be used in the following experiments. All the procedures involving human samples were approved by Taipei Medical University-Joint Institutional Review Board.

### Antibodies

Anti-CD11c-FITC, anti-HLA-DR-FITC, anti-CD-86-PE, anti-DC-SIGN-PE, and anti-CD14-PE were purchased for flow cytometry from eBioscience (San Diego, CA, USA). Anti-DC-SIGN antibody used to block the DC-SIGN receptor was from R&D system (Minneapolis, MN, USA). Anti-ANXA2 antibody was from R&D system or GeneTex (Irvine, CA, USA). Alkaline phosphatase-conjugated goat anti-mouse IgG and goat anti-rabbit IgG were from Bioscience (Taipei, Taiwan, ROC). Human IL-10 and IL-12 antibodies were from PeproTech. Rabbit IgG (H+L) reabsorbed secondary antibody (conjugated DyLight 594) and anti-human IgG (Fc fragment) antibody (conjugated FITC or HRP) were from GeneTex.

### Flow cytometry

To analyze the DC maturation marker HLA-DR and DC-SIGN, we cultured MDDCs at 5×10^4^ per well and stimulated them with 5×105 NPC cells. After 24 h, we collected MDDCs and NPC cells together and washed with PBS containing 1% BSA. They were subsequently incubated with anti-human CD11c-PE antibody (GeneTex) and anti-human HLA-DR-FITC or DC-SIGN-FITC antibody (GeneTex) at 4°C for 1 h, and then washed twice with PBS containing 1% BSA. The stained cells were analyzed with FACSCalibur to determine the number of CD11c and HLA-DR or DC-SIGN double positive cells. Data were processed with CellQuest software.

To detect the DC-SIGN ligand expression on NPC cell membranes, 2×10^5^ NPC cells were collected and washed with PBS containing 0.1% FBS. They were subsequently incubated with the recombinant protein consisting of the extracellular domain of DC-SIGN and the Fc-domain of human IgG1 (DC-SIGN-Fc) recombinant protein (R&D system, USA) at 10 μg/ml for 1 h at room temperature. After incubation, we detected the DC-SIGN-Fc recombinant protein on NPCs with an anti-DC-SIGN antibody.

To determine the major DC-SIGN glycan ligand on NPC cells, 4×10^5^ NPC cells were collected and washed with PBS containing 1% BSA. They were subsequently incubated with 2.5 μg/ml DC-SIGN-Fc protein, 10 mM CaCl_2_ and several concentrations (0, 5, 10, 15, 20, 30, 50, 70 mM) of monosaccharide including mannose and fucose for 1 h at 4°C. After incubation, we detected the DC-SIGN-Fc bound on NPCs with anti-Fc-FITC antibody (GeneTex).

To confirm ANXA2 as a DC-SIGN ligand on NPC cells, 4×10^5^ NPC cells treated with scramble shRNA (scNPC) or an ANXA2 shRNA (shNPC-1 or shNPC-2) were collected and washed with PBS containing 1% BSA. The cells were subsequently incubated with 2.5 μg/ml DC-SIGN-Fc and 10 mM CaCl_2_ at 4°C. After incubation, we detected the DC-SIGN-Fc on NPC cells with anti-Fc-FITC antibody.

### Enzyme-linked immunosorbent assay (ELISA)

Anti-human IL-10 or IL-12 antibody (Invitrogen, USA) was diluted in Coating Buffer A (Invitrogen, USA) to a concentration of 1 μg/ml and used for the detection of NPC mediated IL-10 or IL-12 release from MDDCs. MDDCs were cultured in 24-well plates at a density of 5×10^4^ cells per well. In the DC-SIGN blockage analysis, the MDDCs were pre-treated with 1 μg/ml Anti-DC-SIGN antibody (R&D system, USA) for 1 h. They were then stimulated with 5×10^5^ NPC cells. In ANXA2 blockage analysis, NPC cells were pre-treated with 3 μg/ml Anti-annexin A2 antibody (R&D system, USA) for 1 h before the co-culture.

As suggested in the protocol, 100 μl of diluted anti-human IL-10 or IL-12 antibody was added to individual wells of ELISA plates at 4°C overnight. The capture antibody-coated plates were washed and blocked with Assay Buffer. For comparison, serial diluted standards were added into corresponding wells of microtiter plate along with 100 μl of supernatant from the co-culture and 0.16 μg/ml detecting antibodies. After 2 h of incubation at room temperature, the plate was washed followed by additional diluted avidin-HRP conjugate and incubated for 30 min in room temperature. At the end, Stop Solution was added and the reaction was sit for 30 min in room temperature. Color of each reaction was developed following substrate incubation and measured by Microtiter plate reader at 450 nm with wavelength correction set at 650 nm. To minimize the effects of variation among individual donors, the data were normalized in each experiment as indicated.

### Immunofluorescent staining

NPC cells (10^4^) were seeded on Chamber slides for 2 days. Cells were fixed with 4% paraformaldehyde for 15 min and blocked with 2% BSA on ice for 1 h after washing. DC-SIGN-Fc recombinant protein or IgG at 10 μg/ml was incubated with anti-ANXA2 antibody (1:100 in 1X TBS) in the presence of 10 mM Ca^2+^ or 20 mM EDTA at room temperature for 1 h. Anti-Human IgG (Fc fragment) antibody-FITC or anti-rabbit IgG antibody-DyLight 594 (1:100 in 1X TBS) was added at room temperature for 1 h after washing. Samples were mounted with DAPI at least 30 min. Fluorescence imaging was performed using TCS SP5 Confocal Spectral Microscope Imaging System (Leica, Germany).

### Immunohistochemistry

Formalin-fixed and paraffin-embedded human tissues from three NPC subjects were sectioned in 5 μm and deparaffinized in xylene. Sections were incubated at 120°C in 10 mM citric acid, pH 6 for 20 min for antigen retrieval. Anti-ANXA2 monoclonal antibody (R&D) was applied followed by HRP-conjugated secondary antibody incubation. The sections were developed using 3,3′-Diamino-benzidine hydrochloride (DAB). Counterstaining was performed with hematoxylin before dehydration and mounting. All the procedures involving human samples were approved by Taipei Medical University-Joint Institutional Review Board.

### RNA interference

DC-SIGN siRNA (5′-GGC AAU GGC UGG AAC GAC GAC AAA U-3′) was added into the MDDC culture at day 1. The transfected MDDCs at 2×10^4^ per well were seeded on a 24-well plate and co-cultured with 2×10^5^ NPC for 24 h. The co-cultured media were analyzed for IL-10 concentration.

ANXA2 lentiviral shRNAs (TRCN0000289717 or #1, 5′-GCAGGAAATTAACAGAGTCTA-3′ and TRCN0000289781 or #2, 5′-CGGGATGCTTTGAACATTGAA-3′) and scramble shRNA control (pLAS.Void) with mismatch sequences were purchased from Academia Sinica of Taiwan. They were used for transfection of the packaging HEK 293T cells with helper vectors, using Fugene 6 transfection reagent (Roche). The medium containing lentiviral particles was harvested, filtered, aliquoted and stored at −80°C. These viruses were used to transduce 5×10^5^ NPC cells/well in the presence of 8 μg/ml polybrene (Sigma-Aldrich) in a 24-well plate. Transduced cells were selected in DMEM medium containing 5 μg/ml puromycin (Sigma-Aldrich).

### Preparation of NPC membrane proteins

To prepare NPC membrane proteins, cells at 3~5×10^6^ from a 10-cm dish were washed and incubated with the extraction buffer 1 containing protease inhibitor (membrane protein extraction kit) at 4°C for 15 min. After removing the buffer 1, the cells were incubated with the extraction buffer 2 at 4°C for 30 min. Finally, we collected the supernatant which was enriched in membrane proteins.

### Co-immunoprecipitation

Briefly, 2 μg of DC-SIGN-Fc recombinant protein was preincubated in 100 μl of protein A magnetic beads with continuous end-to-end gentle rotation at 4°C for 2 h. At the end of reaction, magnetic beads were washed with TBST. For precipitation, NPC membrane proteins in the presence of CaCl_2_ or EDTA (as control) were incubated with beads pre-bound DC-SIGN-Fc with continuous end-to-end gentle rotation at 4°C for 2 h. At the end of precipitation, magnetic beads containing complexes of DC-SIGN-Fc and its binding components were eluted by 0.2 M glycine (pH 2.5) to separate the protein from the beads and neutralized in 1 M Tris (pH 8.5). We analyzed the eluted proteins using SDS-PAGE and silver staining (Bio-Rad, USA).

### LC/MS/MS analysis

All mass spectrometric analyses were performed using an LTQ-Orbitrap (Discovery) hybrid mass spectrometer with a nanoelectrospray ionization source (ThermoElectron, San Jose, CA, USA) coupled to a nano-flow high-performance liquid chromatography (HPLC) system (Agilent Technologies 1200 series, Germany). An Agilent C18 column (100_0.075mm, 3.5 mm particle diameter) with mobile phases of A (0.1% formic acid in water) and B (0.1% formic acid in acetonitrile) was used. The pump flow rate was set at 0.5 mL/min, and peptide elution was achieved using a linear gradient of 5–35% B for the first 30 min followed by a rapid increase to 95% B over the next 10 min. The conventional MS spectra (Survey Scan) were acquired at high resolution (M/DM, 60,000 full width half maximum) over the acquisition range of m/z 200–2000 and a series of precursor ions were selected for the MS/MS scan. The former examined the accurate mass and the charge state of the selected precursor ion, while the latter acquired the spectrum (CID spectrum m or MS/MS spectrum) for the fragment ions generated by collision-induced dissociation.

The mass spectrometry dataset was analyzed using Xcalibur software (version 2.0 SR1). Product ion scans obtained from the MS/MS experiments were investigated using the database search software SEQUEST (TURBO).

### Glycan digestion assay

For peptide-N-glycosidase (PNGase) F (New England Biolabs, USA) treatment, membrane proteins (180 μg) were prepared from NPC cells and suspended in 25 mM Tris and 150 mM NaCl with or without 50,000 U/ml PNGase F at 37°C for 2 h. After digestion membrane proteins were precipitated with DC-SIGN-Fc, followed by SDS-PAGE and immunoblotting with anti-annexin A2 or anti-DC-SIGN antibody.

### Reverse transcriptase quantitative polymerase chain reaction (RT-qPCR)

Total RNAs (1 μg) were reversely transcribed using oligo-dT primers and GoScript™ Reverse Transcription System (Promega, life sciences). Quantitative PCR was performed using KAPA SYBR® FAST qPCR Kit in a LightCycler® 480 (Roche) according to the manufacturer's protocol. The PCR samples were incubated 1 min at 50 °C followed by 1 min at 95 °C and 40 cycles of 5 s at 95 °C and 30 s at 60 °C. The dissociation reaction was performed as 1 min at 95 °C and 30 s at 55 °C. The primer sequences used are 5′-CTCTACACCCCCAAGTGCAT-3′ (Forward) and 5′-TCAGTGCTGATGCAAGTTCC-3′ (Reverse) for ANXA2; 5′-CAGCAAGAGCACAAGAGGAAG-3′ (Forward) and 5′-TGGTACATGACAAGGTGCGG-3′ (Reverse) for glyceraldehyde 3-phosphate dehydrogenase (GAPDH) as a control.

### Western blotting

Proteins from SDS-PAGE were transferred to PVDF membranes at 100 V/400 mA for 2 h, and blocked at room temperature for 1 h. After blocking, the membrane was incubated in 1% BSA/PBS with the anti-annexin A2 (R&D system, USA) or anti-DC-SIGN (GeneTex, USA) primary antibody in a 1:5,000 dilution with gentle shaking at 4°C overnight. After washed for three times with PBST, the membrane was incubated with the HRP-conjugated anti-mouse (GeneTex) or rabbit IgG (GeneTex) secondary antibody in a 1:10,000 dilution for 2 h. After the secondary antibody incubation, the membrane was washed 3 times with PBST. For visualization, the membrane was treated with Super-Signal West Pico Chemiluminescent kit (Thermo Fisher Scientific) and imaged.

### Statistical methods

Each experiment was performed at least three times. SigmaPlot software and the Student t test were used for statistical analysis. A value of p < 0.05 was considered statistically significant. Results are shown as mean ± standard deviation.
